# The N-Terminal DH-PH Domain of Trio Induces Cell Spreading and Migration by Regulating Lamellipodia Dynamics in a Rac1-Dependent Fashion

**DOI:** 10.1371/journal.pone.0029912

**Published:** 2012-01-06

**Authors:** Jos van Rijssel, Mark Hoogenboezem, Lynn Wester, Peter L. Hordijk, Jaap D. Van Buul

**Affiliations:** Department of Molecular Cell Biology, Sanquin Research and Landsteiner Laboratory, Academic Medical Center, University of Amsterdam, Amsterdam, The Netherlands; Virginia Tech, United States of America

## Abstract

The guanine-nucleotide exchange factor Trio encodes two DH-PH domains that catalyze nucleotide exchange on Rac1, RhoG and RhoA. The N-terminal DH-PH domain is known to activate Rac1 and RhoG, whereas the C-terminal DH-PH domain can activate RhoA. The current study shows that the N-terminal DH-PH domain, upon expression in HeLa cells, activates Rac1 and RhoG independently from each other. In addition, we show that the flanking SH3 domain binds to the proline-rich region of the C-terminus of Rac1, but not of RhoG. However, this SH3 domain is not required for Rac1 or RhoG GDP-GTP exchange. Rescue experiments in Trio-shRNA-expressing cells showed that the N-terminal DH-PH domain of Trio, but not the C-terminal DH-PH domain, restored fibronectin-mediated cell spreading and migration defects that are observed in Trio-silenced cells. Kymograph analysis revealed that the N-terminal DH-PH domain, independent of its SH3 domain, controls the dynamics of lamellipodia. Using siRNA against Rac1 or RhoG, we found that Trio-D1-induced lamellipodia formation required Rac1 but not RhoG expression. Together, we conclude that the GEF Trio is responsible for lamellipodia formation through its N-terminal DH-PH domain in a Rac1-dependent manner during fibronectin-mediated spreading and migration.

## Introduction

Cell adhesion and spreading on extracellular matrix proteins such as fibronectin (FN) is indispensable for many important physiological processes, such as development, growth and migration. During cell spreading, the actin cytoskeleton is regulated by Rho-GTPases. These Rho-GTPases serve as molecular switches, transducing signals from the extracellular environment to elicit cellular responses, such as changes in morphology and directional migration [1]. Rho-GTPase family members are small proteins that cycle from an inactive, GDP-bound to an active GTP-bound state. When bound to GTP, they interact with a broad range of downstream effectors, initiating intracellular signals. The exchange from GDP to GTP is mediated by enzymes called Guanine nucleotide Exchange Factors (GEFs). These regulate local activation of GTPases and thereby control the downstream effects of these GTPases [2].

Among the 22 known Rho-GTPase proteins, RhoA stimulates the formation of stress fibers [3], whereas Rac1 is known to induce membrane ruffling and lamellipodia formation [4]. Upon integrin-mediated adhesion to fibronectin-coated surfaces, Rac1 is activated, resulting in membrane ruffling and cell spreading [5]. Rac1 activation during cell spreading was claimed to be regulated by a close family member of Rac1, RhoG, through its activation of the bipartite ELMO and Dock180 GEF complex [6], [7]. However, other investigators showed that nearly complete RhoG depletion did not substantially inhibit cell adhesion, spreading, migration or Rac1 activation [8]. We have previously shown that Rac1 activity and effector functions can also be regulated through its hypervariable C-terminal tail by binding partners, such as the GEF β-Pix and caveolin-1 [9], [10]. Activation of Rac1 by the GEF β-Pix appeared to be dependent on the direct interaction between a proline-rich region within the Rac1 C-terminus and the SH3 domain that precedes the Dbl-homology/Pleckstrin-homology (DH-PH) GEF domain of β-Pix. The presence of SH3 domains adjacent to the DH-PH domain is commonly observed in GEFs that are specific for Rho-family GTPases [11]. However, whether the interaction of the Rac1 C-terminus with SH3-domains in these GEFs represents a general prerequisite for Rac1 activation remains to be established.

The GEF Trio contains two DH-PH domains of which the N-terminal DH-PH domain has been shown to activate Rac1 and RhoG [12], [13]. The second, C-terminal DH-PH domain is known for its specific exchange of GTP on RhoA (Medley et al., 2000). Trio also contains two SH3 domains, of which only one is located in close proximity of the N-terminal DH-PH domain. It has been reported that overexpression of the N-terminal GEF domain of Trio including the SH3 domain promotes 3T3 cell spreading and haptotactic migration towards a fibronectin gradient [14]. Moreover, it was shown that Trio mediated the migration of granule cells during cerebellum development [15]. In malignant glioma's, Trio-mediated Rac1 activation was implicated in cell migration and invasion [16], suggesting involvement of the N-terminal GEF domain of Trio. Interestingly, the N-terminal Trio DH-PH domain is 3 times more efficient in exchanging GTP on RhoG than on Rac1 [17]. Using a dominant-negative construct of RhoG, Blangy and co-workers could block Trio-D1-mediated Rac1 activation, suggestive for a role for RhoG, upstream of Rac1 [12].

In this study, we demonstrate that the N-terminal GEF domain of Trio can interact with the C-terminal hypervariable domain of Rac1, but not of RhoG, in an SH3-domain dependent manner. The SH3 domain is, however, dispensable for Trio-mediated Rac1 and RhoG activation. Using siRNA-mediated silencing of RhoG expression, we show that Trio-induced Rac1 activation is also independent of RhoG. In Trio-shRNA expressing HeLa cells, Rac1 activation and cell spreading was impaired, whereas active RhoG levels did not change. The impaired cell spreading and migration were rescued by expression of the N-terminal DH-PH domain of Trio. Moreover, using kymograph analysis, we show that the N-terminal DH-PH domain of Trio mediates lamellipodia dynamics. Using siRNA technique to silence Rac1 or RhoG in Trio-deficient cells that were rescued with the N-terminal DH-PH domain of Trio, we made clear that Trio-induced lamellipodia dynamics was dependent on Rac1 and not RhoG. These data show that Trio regulates cell spreading and migration through its N-terminal DH-PH domain in a Rac1-dependent manner.

## Results

**Figure 1:**
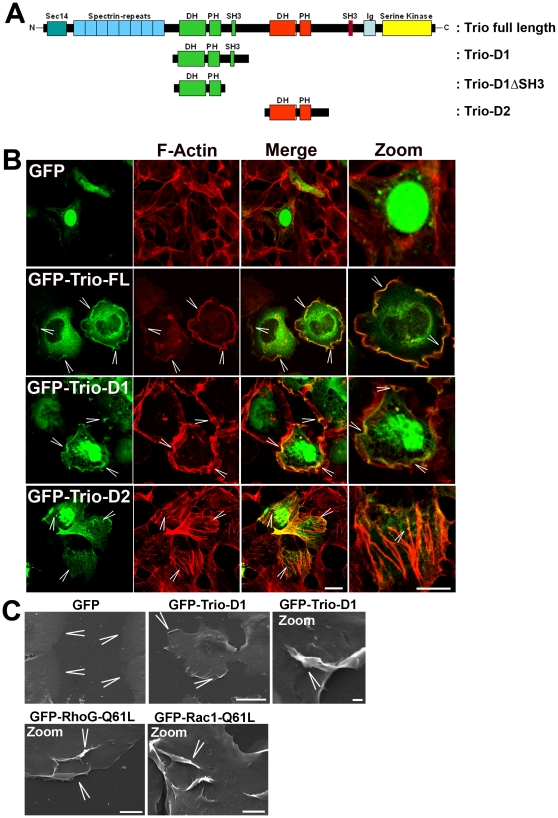
Trio induces membrane ruffles. (**A**) Schematic overview of the Trio protein (3097 amino acids, molecular weight approximately 350 kDa), indicating in green the N-terminal DH-PH unit including an SH3 domain and in red the C-terminal DH-PH unit. The third catalytic domain of Trio is a kinase domain (yellow). At the N-terminus, a Sec14 domain and spectrin repeats are present. Below the GFP/Myc-tagged constructs used in this manuscript are depicted: Trio-D1 encodes for the N-terminal DH-PH domain including the flanking SH3 domain, Trio-D1ΔSH3 domain represents the N-terminal DH-PH domain lacking the SH3 domain, and Trio-D2 representing the C-terminal DH-PH domain. (**B**) HeLa cells were cultured on glass cover slips and transfected as indicated with GFP-tagged constructs. Immunofluorescent imaging showed that GFP did not affect the morphology of the cells. GFP-Trio full length (FL) and GFP-Trio-D1 induced lamellipodia (arrowheads) and co-localized with F-actin (red), as is shown in the merge images. For the Trio-FL, 68%±7 of the transfected cells induced lamellipodia as illustrated in figure 1B. For Trio-D1, 79%±4 of the transfected cells induced lamellipodia. GFP-Trio-D2 in green induced stress fibers (arrowheads), shown by F-actin staining in red. 46%±12 of these transfected cells i9nduced stress fibers, as shown. Data are mean ± SEM. Bar, 20 µm. Images at the right show merged magnification of F-actin in red and GFP-tagged protein in green. Bar, 10 µm. (**C**) Changes in morphology analyzed by scanning electron microscopy. No change in morphology is observed at the periphery or surface of GFP-expressing HeLa cells (arrowheads), whereas Trio-D1 induced large dorsal and lateral lamellipodia (arrowheads). Bar, 50 µm. Image on the right shows a magnification (Zoom) of Trio-D1-induced dorsal lamellipodia (arrowheads). Bar, 5 µm. Two lower images show lamellipodia (arrowheads), induced by a constitutively active form of RhoG (Q61L) (left image) and Rac1 (Q61L) (right image), both comparable with the lamellipodia induced by Trio-D1. Bar, 10 µm.

Trio contains two DH-PH domains, schematically represented in figure 1A. We found that expression in HeLa cells of a GFP-tagged N-terminal DH-PH domain, hereafter indicated as Trio-D1, induced lamellipodia (Figure 1A and 1B), which underscored data reported by Blangy and co-workers [12]. Also, increased cortical actin is observed in cells transfected with Trio-D1, which resembled a phenotype that is normally observed for activated Rac1 [10]. Expression of the C-terminal DH-PH domain, hereafter indicated as Trio-D2, induced the formation of actin stress fibers, which is in line with previous reports, showing that Trio-D2 facilitates the exchange of GDP for GTP on RhoA [18], [19]. Expression of full-length Trio resulted in a phenotype that resembled that of Trio-D1, i.e. the induction of lamellipodia (Figure 1A). Using scanning electron microscopy, we detected Trio-D1-induced membrane ruffling on the surface of the cells. These experiments also confirmed that Trio-D1-induced morphological changes (i.e. apical and lateral membrane ruffles) were similar to those induced by constitutively active RhoG-Q61L or Rac1-Q61L (Figure 1C).

**Figure 2:**
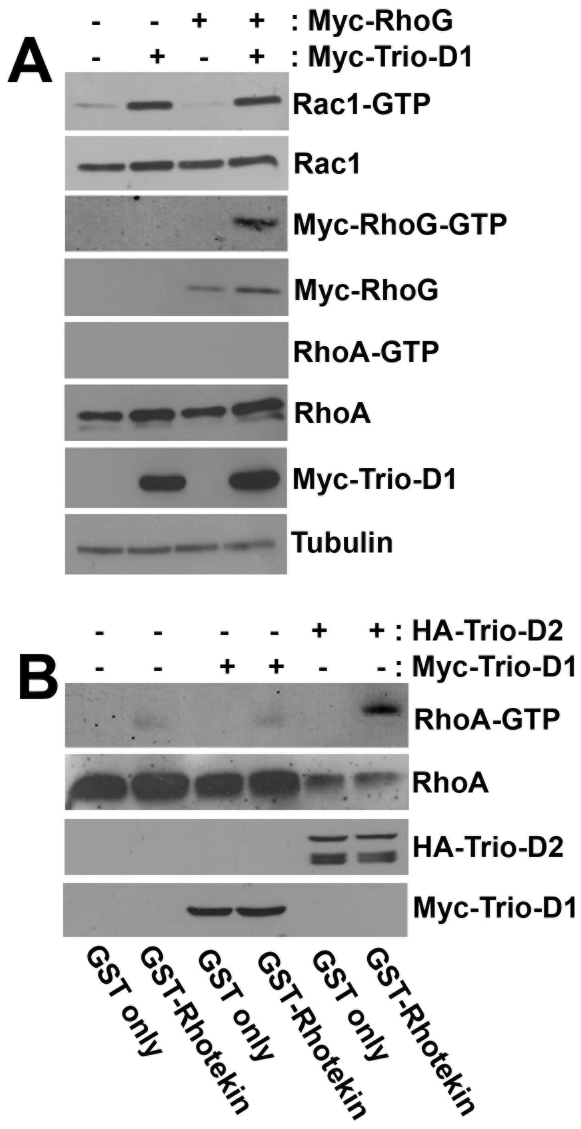
Trio-D1 activates Rac1 and RhoG, but not RhoA. (**A**) HeLa cells were transfected with Myc-Trio-D1 and Myc-RhoG as indicated. Rac1-GTP and RhoG-GTP and RhoA-GTP levels were measured as described in Materials and Methods and show that Trio-D1 activates RhoG and Rac1, but not RhoA. Tubulin is shown as protein loading control. (**B**) HeLa cells were transfected with HA-Trio-D2 and Myc-Trio-D1 as indicated. RhoA-GTP was measured as described in Materials and Methods and shows that Trio-D2, but not Trio-D1 activates endogenous RhoA. Second panel from above shows RhoA protein loading in the cell lysates. Data are representative for at least three independent experiments.

We next measured whether exogenous Trio-D1 or Trio-D2 were able to activate Rac1, RhoG or RhoA, using biochemical pull-down assays, as described in Materials and Methods. The data showed that Trio-D1 activated endogenous Rac1, as well as exogenous Myc-RhoG (Figure 2A). As expected, Trio-D1 did not activate RhoA. As a control, we confirmed Trio-D1-induced JNK activation (data not shown), as was also shown by Bellanger and co-workers [18]. Additional experiments with GST-Rhotekin showed that the expression of Trio-D2 increased the levels of endogenous GTP-bound RhoA (Figure 2B)

**Figure 3:**
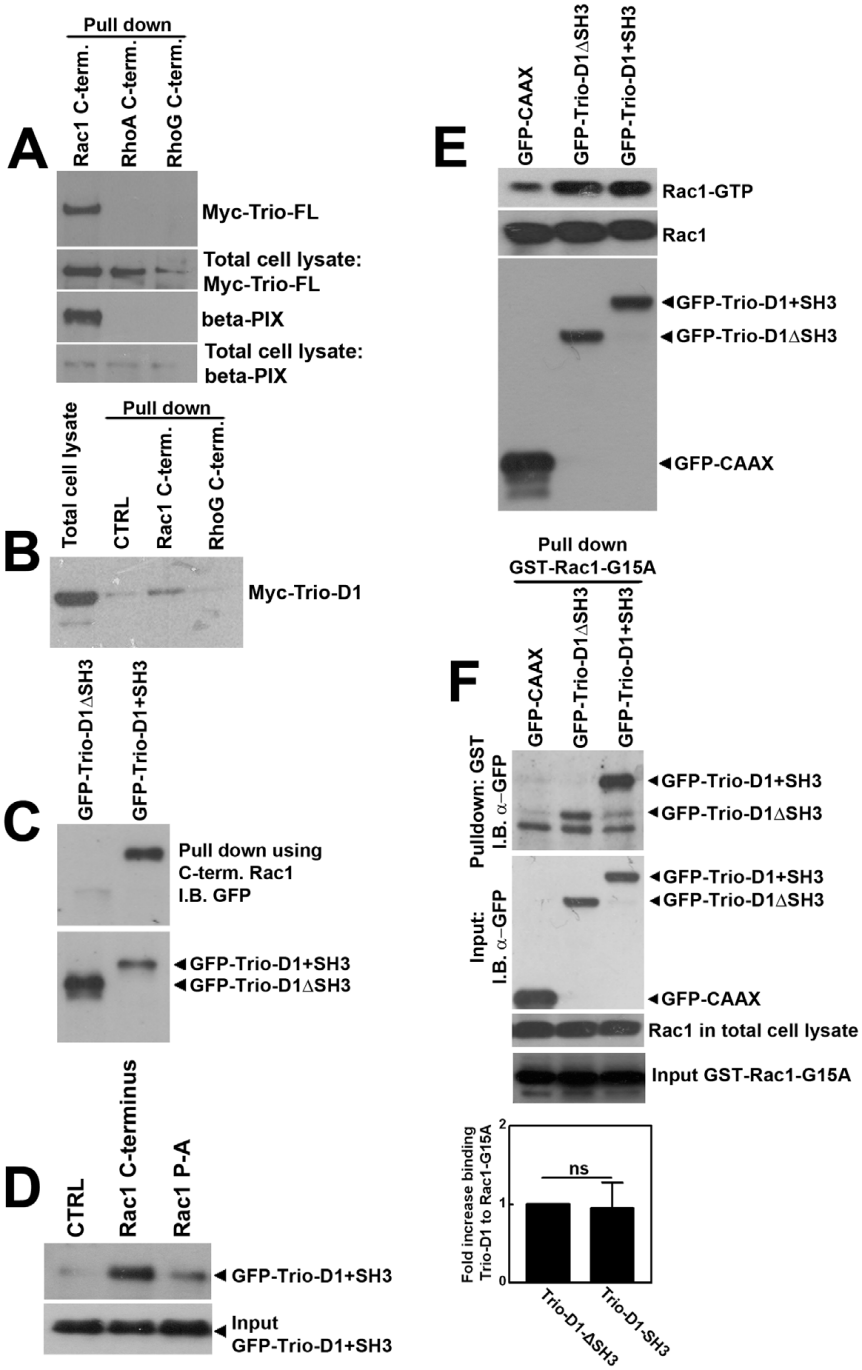
Trio-D1 binds to the C-terminus of Rac1 but not of RhoG and activates Rac1 independent of its SH3 domain. (**A**) HeLa cells were transfected with Myc-Trio-Full-length (FL) and a pull-down experiment with biotin-tagged peptides that encode for the last 10 amino-acids of the C-terminus of Rac1, RhoG and RhoA was performed, as described in Materials and Methods. Western blot analysis showed that Trio-FL binds to the Rac1 C-terminus peptide, but not to RhoA or RhoG C-termini. As a control, β-Pix binding to the C-terminus of Rac1, but not of RhoA or RhoG is shown. (**B**) Myc-Trio-D1 was transfected into HeLa cells, and a peptide pull down was performed as described under A. Western blot analysis showed that Trio-D1 associates with the C-terminus of Rac1, but not with the CTRL or RhoG peptide. Left lane shows Myc-Trio-D1 input. (**C**) HeLa cells were transfected with GFP-Trio-D1ΔSH3 or GFP-Trio-D1+SH3 constructs and a Rac1 C-terminal peptide pull down was performed. Western blot analysis showed that the C-terminus of Rac1 required the SH3 domain of Trio-D1 to interact. Blots were incubated with an Ab against GFP to stain for Trio constructs. (**D**) HeLa cells were transfected with GFP-Trio-D1+SH3 constructs and a peptide pull down was performed with biotinylated peptides encoding control sequence, the Rac1 C-terminal domain or the Rac1 C-terminal domains in which the proline stretch had been mutated to alanines (P/A) [10]. Western blot analysis showed that Trio-D1+SH3 required the proline-rich stretch in the Rac1 C-terminus to bind. Blots were incubated with an Ab against GFP to stain for Trio constructs. (**E**) HeLa cells were transfected with GFP-CAAX, GFP-Trio-D1ΔSH3 or GFP-Trio-D1+SH3 constructs, and Rac1-GTP activity assays were performed as described in Materials and Methods. Western blot analysis showed that Rac1 is activated by Trio-D1, independent of the SH3 domain (upper panel). (**F**) HeLa cells were transfected with GFP-CAAX, GFP-Trio-D1ΔSH3 or GFP-Trio-D1+SH3 constructs and a pull-down assay using glutathione-beads to precipitate GST-Rac1-G15A mutants was performed as described in Materials and Methods. Western blot analysis showed that Rac1 needed the SH3 domain of Trio-D1 to interact (upper panel), because the binding was less efficient when Trio-D1 lacked the SH3 domain. Lower panel shows GST-Rac1-G15A input. Lower unidentified band in upper panel is due to GST isolation and a-specific staining of the antibody. Graph below shows the quantification of the binding of GST-Rac1-G15A to Trio-D1ΔSH3 and Trio-D1+SH3. No significant difference was found for the presence of the SH3 domain in the binding to GST-Rac1-G15A. Experiment was carried out three times, independently from each other. Data are mean ± SEM. ns: not significant.

Activation and targeting of Rac1 by the GEF β-Pix depends on the direct interaction of the Rac1 hypervariable C-terminus with the SH3-domain of β-Pix [9]. Using a biotinylated peptide comprising the Rac1 C-terminus, we observed that full-length Myc-Trio also interacted with the Rac1 C-terminus, whereas Myc-Trio did not bind to the C-terminus of RhoG (Figure 3A). Since the N-terminal DH-PH domain of Trio activated Rac1, we tested whether Trio-D1 could also interact with the hypervariable Rac1 C-terminus. The results showed that Myc-tagged Trio-D1 interacted with the Rac1 C-terminal peptide, but not with the RhoG C-terminal peptide (Figure 3B). To study the involvement of the flanking SH3 domain of Trio-D1 in the interaction with the Rac1 C-terminus, we generated Trio-D1 mutants that lacked the SH3 domain (Trio-D1ΔSH3; Figure 1A). These experiments showed that the SH3 domain of Trio-D1 was required for the binding to the Rac1 C-terminus (Figure 3C). A peptide in which the proline-rich region of the Rac1 C-terminus was replaced with alanines (Rac1 P-A) showed a reduced interaction with Trio-D1 (Figure 3D). These data confirmed that Trio-D1 interacted with the Rac1 C-terminal hypervariable domain through the proline-rich stretch in a SH3-domain-dependent manner.

To determine whether the interaction of the Rac1 C-terminus with the SH3 domain of Trio-D1 is a prerequisite for Rac1 activation by Trio-D1, we measured Rac1 activation by Trio-D1 lacking its SH3 domain. Overexpression of GFP-Trio-D1 induced an increase in Rac1 activation compared to control membrane-anchored GFP (GFP-CAAX) transfected cells. However, deletion of the SH3 domain did not affect the ability of Trio-D1 to activate Rac1 (Figure 3E). Analysis of the interaction of Trio-D1 and Trio-D1ΔSH3 with a Rac1 mutant that can no longer bind the nucleotides GDP or GTP (GST-Rac1-G15A) and therefore has a high affinity for GEFs [20] showed no increase in binding for Trio-D1 containing the SH3 domain compared to the one lacking the SH3 domain to nucleotide-free Rac1 (Figure 3F). These results therefore suggest that the SH3 domain is not required for Trio-D1-mediated nucleotide exchange on Rac1.

**Figure 4:**
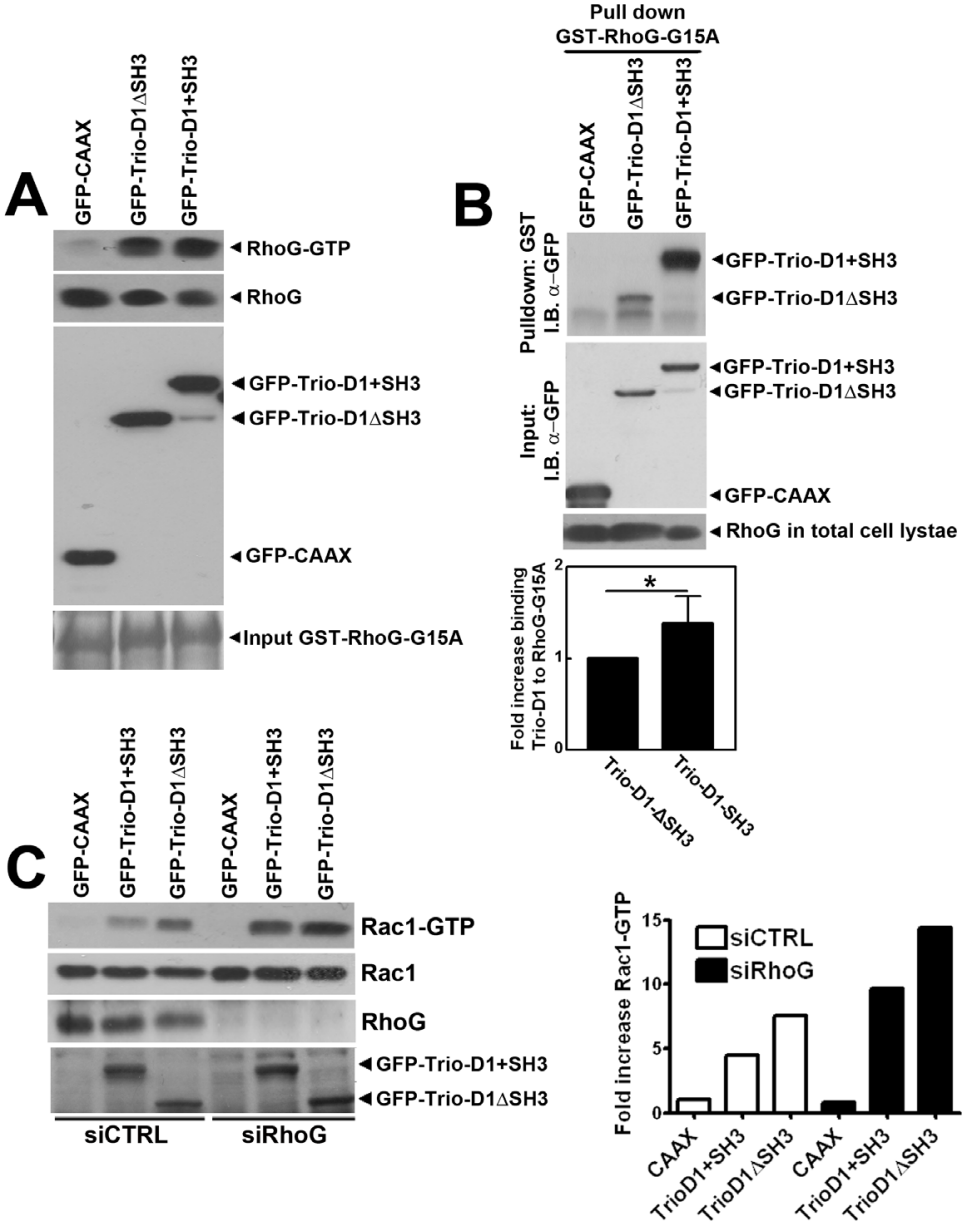
Trio-D1 activates RhoG independent of its SH3 domain. (**A**) HeLa cells were transfected with GFP-CAAX, GFP-Trio-D1ΔSH3 or GFP-Trio-D1+SH3 constructs, and RhoG-GTP activity assays were performed as described in Materials and Methods. Western blot analysis showed that RhoG is activated by Trio-D1, independent of the SH3 domain (upper panel). Middle panel showed the expression of endogenous RhoG in HeLa cells. Lower panel shows the expression of the Trio constructs. (**B**) HeLa cells were transfected with GFP-CAAX, GFP-Trio-D1ΔSH3 or GFP-Trio-D1+SH3 constructs, and a pull-down assay using glutathione-beads to precipitate GST-RhoG-G15A mutants was performed as described in Materials and Methods. Western blot analysis showed that RhoG required the SH3 domain of Trio-D1 to interact (upper panel), although the binding was less efficient when Trio-D1 lacked the SH3 domain. Middle panels show the expression of constructs in total cell lysates using an Ab against GFP and the lower panels show the loading control for RhoG in total cell lysates and GST-RhoG-G15A input. Lower unidentified band in upper panel is due to GST isolation and a-specific staining of the antibody. Graph below shows the quantification of the binding of GST-RhoG-G15A to Trio-D1ΔSH3 and Trio-D1+SH3. A significant difference is found for the presence of the SH3 domain in the binding to GST-RhoG-G15A. Experiment is carried out three times, independently from each other. Data are mean ± SEM. * p<0.05. (**C**) HeLa cells were transfected with RhoG siRNA. Rac1-GTP levels were measured as described in Materials and Methods and show that GFP-Trio-D1 activates Rac1 independently from RhoG. In fact, Trio-D1-induced Rac1 activity was increased when RhoG was silenced. Middle panel shows Rac1 protein for loading control in cell lysates and lower panels show the expression of endogenous RhoG protein and the different Trio-D1 constructs in cell lysates. Graph on the right shows the quantification of the band intensities, measured using ImageJ software and show that RhoG silencing increased TrioD1-induced Rac1-GTP levels 2-fold.

Using a dominant-negative RhoG construct, Blangy and co-workers have suggested that Trio-D1-mediated Rac1 activation is dependent on RhoG [12]. Analysis of RhoG activation using ELMO as bait showed that the SH3 domain is not required for Trio-D1-induced RhoG activation (Figure 4A). Binding of Trio-D1 lacking the SH3 domain to a nucleotide-free RhoG mutant (GST-RhoG-G15A) was significantly less efficient than Trio-D1+SH3 (Figure 4B). These data indicate that the SH3 domain may promote, but is not necessarily required for Trio-D1-mediated nucleotide exchange on RhoG. To determine whether RhoG is involved in Rac1 activation by Trio-D1, we used RhoG-specific siRNA to silence RhoG expression in HeLa cells (Figure 4C). These results show that Rac1 activation by Trio-D1 and Trio-D1ΔSH3 is not impaired, but rather increased in RhoG knockdown cells (Figure 4C). Rac1 activation by the N-terminal GEF domain of Trio is therefore independent of SH3 domain-mediated interactions and RhoG expression.

**Figure 5:**
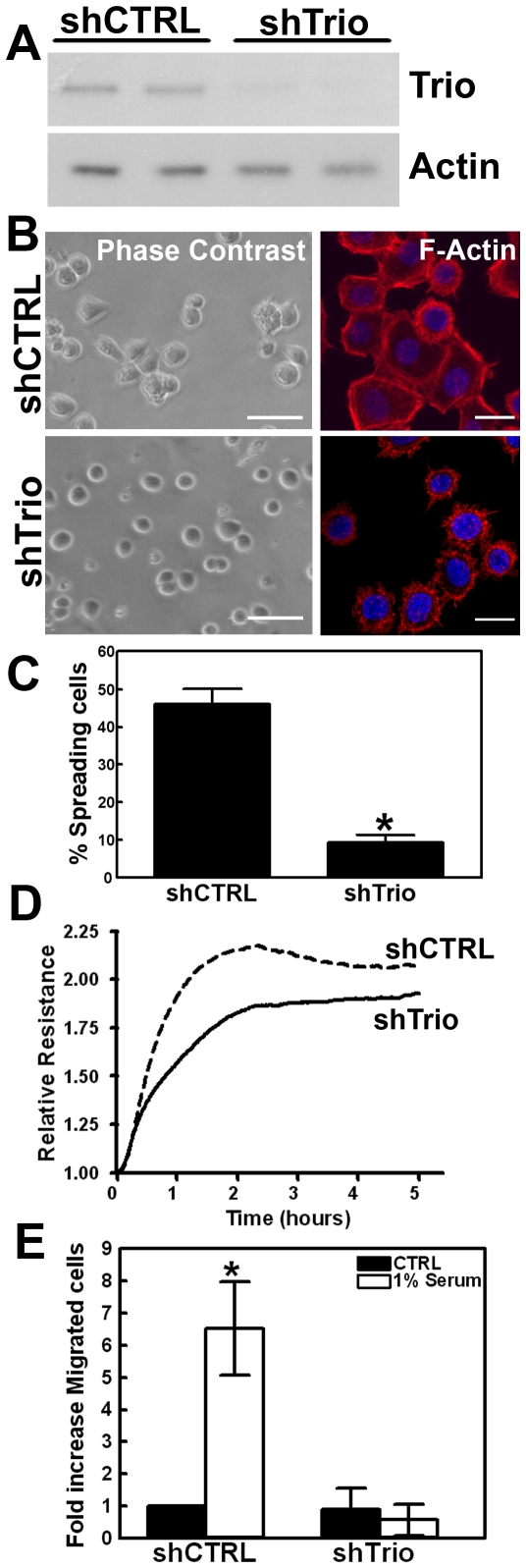
Silencing Trio results in impaired FN-mediated cell spreading. (**A**) shRNA against Trio reduces endogenous Trio expression in HeLa cells, whereas shCTRL (scrambled sequence) did not affect Trio expression. Two independent cell line clones were tested. Actin is shown as protein loading control. (**B**) Cells were allowed to spread for 3 hours under serum-free conditions on FN (10 µg/ml). Phase contrast shows spreading cells in shCTRL-treated cells, whereas shTrio-treated cells display a round phenotype. Bar, 100 µm. F-actin staining in red underscores the defect in spreading of the shTrio-treated cells. Nuclei are in blue. Bar, 20 µm. (**C**) Percentage of cells that spread was quantified. A spreading cell was positively scored when the phenotype resembled the cell shown in figure 5B, right upper image. Experiment was repeated five times in triplicate. Data are mean ± SEM. *p<0.01. (**D**) Cells were plated on FN-coated gold electrodes and spreading was measured in time as indicated. ShTrio-treated cells (solid line) showed reduced spreading capacity compared to shCTRL-treated cells (dashed line). Per array, 200,000 cells were plated. (**E**) In a Transwell system, migration was measured. Filters were coated with FN and HeLa cells (shCTRL or shTrio) were added to the upper compartment and allowed to migrate for 5 h to 1% serum, which was present in the lower compartment. Number of cells that migrated across the filter was counted by nuclei staining and the migration of shCTRL HeLa cells was set to 1. Migration across a FN-coated filter is reduced upon Trio silencing. Experiment was repeated four times in duplicate. Data are mean ± SEM. *p<0.01.

To show the functional significance of the Trio-D1 GEF domain within full-length Trio, we used HeLa cells, stably expressing a Trio shRNA (Figure 5A). Stable knockdown of Trio resulted in a severe defect in the ability of the cells to spread on fibronectin and most of the shTrio cells remained round (Figure 5B). Quantification of spreading cells showed that after 3 hours almost 50% of the shCTRL cells formed lamellipodia and spread on fibronectin, whereas in shTrio cells only 10% of the cells was able to do so (Figure 5C). In addition, quantitative analysis of cell spreading on fibronectin-coated gold electrodes using electrical cell-substrate impedance sensing (ECIS) technology [9], [10] showed less efficient spreading of Trio-deficient cells compared to control cells (Figure 5D). Moreover, Trio knockdown cells were severely impaired in their migratory capacity towards 1% (v/v) FCS across fibronectin-coated Transwell filters (Figure 5E).

**Figure 6:**
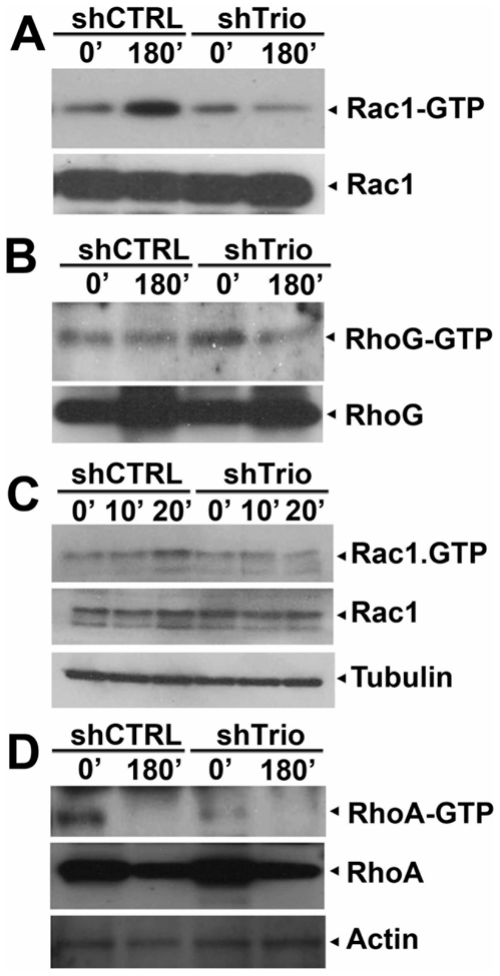
Trio regulates Rac1 activity upon FN-mediated cell spreading. (**A**) Rac1 activity was measured with biotin-CRIB peptides as described in Materials and Methods. Rac1 activity was increased after 3 h of spreading in shCTRL cells, whereas changes in Rac1 activation were absent in Trio-deficient cells (shTrio). (**B**) Trio RhoG activity was measured with GST-ELMO as bait (Materials and Methods). RhoG activity was unaltered in shCTRL and shTrio cells upon cell spreading on FN. (**C**) Early Rac1 activation upon spreading was affected in Trio-deficient cells as well. Rac1-GTP levels were measured upon 10 or 20 minutes spreading on FN or in suspension as described under A. (**D**) RhoA activity was measured in the GST-Rhotekin pull-down assay as described in Materials and Methods. RhoA activity was high in cells that were in suspension (0 minutes) and decreased upon spreading on FN (180 minutes). No difference between shCTRL and shTrio cells was measured. All experiments described above were carried out at least three times.

Analysis of Rac1 and RhoG activation during HeLa cell spreading demonstrated only induction of Rac1 activity (Figure 6A), whereas no detectable increase in RhoG activation was detected (Figure 6B). However, in Trio shRNA cells, no induction in Rac1 activity was measured (Figure 6A), suggesting that the cell spreading is mainly due to Rac1 activation by Trio. Del Pozo and co-workers showed that Rac1 is also activated upon early spreading. Rac1 was activated upon 20 minutes of spreading on FN [5]. However, in Trio-deficient cells, Rac1-GTP levels were decreased, indicating for Trio involvement in Rac1-mediated early spreading events (Figure 6C). In contrast to Rac1, RhoA is known to be temporarily inactivated during cell spreading (Figure 6D). Although RhoA activity in shTrio HeLa cells in suspension was lower than in control cells, after 3 hours of cell spreading no RhoA activation was measured in both control and Trio-silenced cells (Figures 6D and S1).

**Figure 7:**
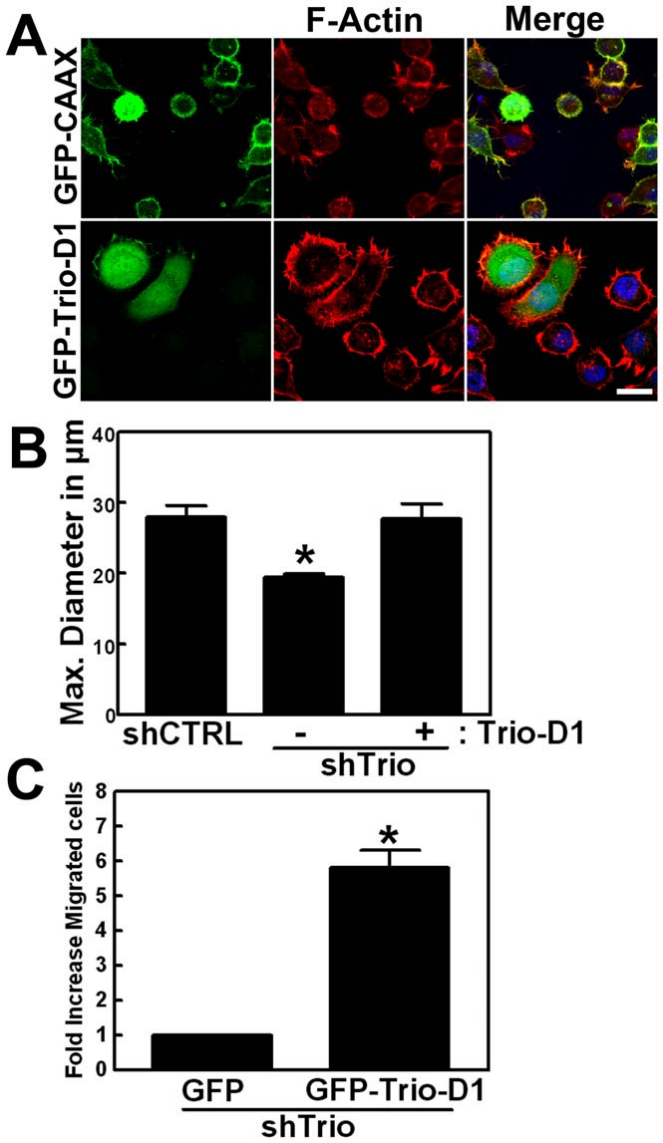
Trio-D1 rescues the spreading and migration defect in Trio-deficient cells. (**A**) GFP-CAAX (upper panels) or GFP-Trio-D1+SH3 (lower panels) were transfected into Trio-deficient cells. Next, cells were allowed to spread for 3 h on FN. GFP-tagged proteins are visible in green, F-actin in red and the merge is shown in yellow. Nuclei are in blue. Bar, 20 µm. (**B**) Graph shows the quantification of cell spreading on FN. The maximum diameter of the cell was measured in µm and displayed on the Y-axis. Experiment was carried out four times in duplicate. Data are mean ± SEM. *p<0.01. (**C**) Bar graph shows quantification of cell migration assay across FN-coated Transwell filters. Trio-deficient HeLa cells were transfected with GFP alone or GFP-Trio-D1 and allowed to migrate from the top to the bottom part of the filter. The lower compartment contained 1% serum. Number of cells that migrated across was counted by nuclei staining and GFP-expressing cells counted were set as 1. Experiment was carried out four times in duplicate. Data are mean ± SEM. *p<0.001.

Since the shRNA construct to silence Trio is targeted to the N-terminus and does not interfere with the expression of the Trio-D1 domain (see Materials and Methods), Trio-D1 can be tested for rescue of cell spreading. The results showed that Trio-D1 expression, in Trio-deficient cells restored cell spreading in contrast to Trio-deficient cells that were transfected with GFP-CAAX (Figure 7A). Expression of Trio-D2 in Trio-deficient cells did not rescue the impaired cell spreading on FN (Figure S2A). Quantification of cell spreading by measuring the length of the cell body along the longitudinal axis showed that Trio-D1 significantly restored the defect in cell spreading, induced by Trio silencing, to the same extent as that of control HeLa cells (Figure 7B). Moreover, the inability of Trio-deficient cells to migrate across fibronectin-coated filters was recovered when GFP-Trio-D1 was expressed (Figure 7C).

**Figure 8:**
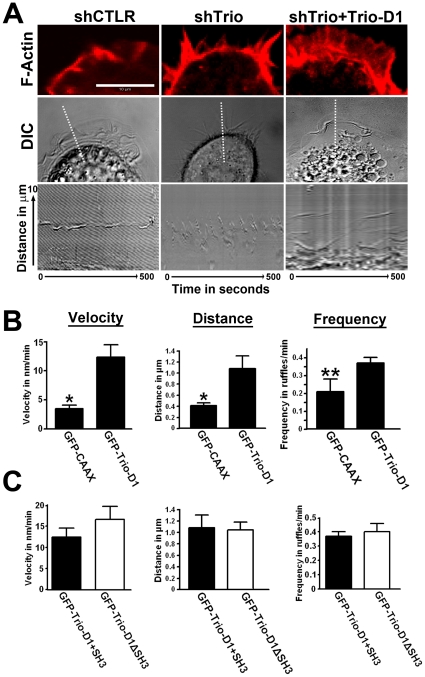
Trio-D1 regulates the dynamics of lamellipodia. (**A**) Upper panel shows magnification of lamellipodia, stained for F-actin in red in cells transfected with scrambled shRNA (shCTRL), shRNA against Trio (shTrio) or Trio-deficient cells transfected with Trio-D1 (shTrio+Trio-D1). Bar, 10 µm. Middle panel shows a still image of a movie showing lamellipodia dynamics in DIC. Dotted line represents the position of the kymograph that is shown in the lower panel. Kymographs represent the dynamics of lamellipodia for 500 seconds as indicated. Y-axis represents 10 µm distance. (**B**) Kymograph analysis as described in Material and Method section of lamellipodia in Trio-deficient HeLa cells was performed. Cells were transfected with GFP-CAAX or GFP-Trio-D1. Trio-D1 promotes the velocity (left graph), distance (middle graph) and frequency (right graph) of lamellipodia dynamics significantly. At least nine different lamellipodia were quantified in nine different cells over three independent experiments. Data are mean ± SEM. *p<0.01; **p<0.01 (**C**) Kymograph analysis as described above. Trio-deficient HeLa cells were transfected with Trio-D1 containing the SH3 domain (closed bars) or lacking the SH3 domain (open bars). No difference was observed between the two conditions in the velocity (left graph), distance (middle graph) or frequency (right graph) of lamellipodia. At least nine different lamellipodia were quantified in nine different cells over three independent experiments. Data are mean ± SEM.

**Figure 9:**
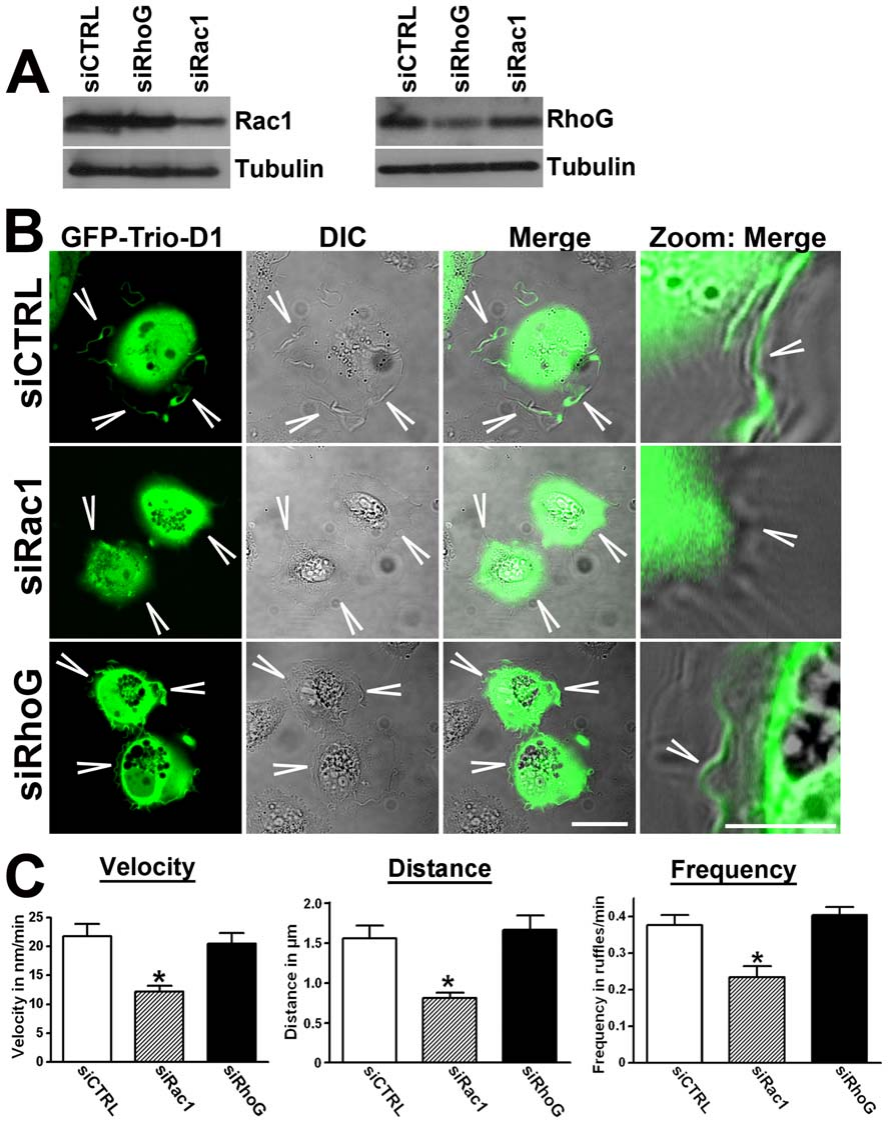
Rac1 is required for Trio-D1-induced lamellipodia. (**A**) Trio-deficient cells were transfected with Trio-D1 and silenced as indicated with siRNA duplexes, as described in Materials and Method section. Tubulin serves as a protein loading control. (**B**) GFP-Trio-D1 is expressed in Trio-deficient HeLa cells and shows lamellipodia formation in siCTRL and siRhoG-treated cells (arrowheads), whereas siRac1-treated cells showed impaired Trio-D1-induced lamellipodia formation (arrowheads). Bar, 20 µm. Images at the right show magnification area. Bar, 5 µm. (**C**) Kymograph analysis as described in Materials and Methods section of the dynamics of lamellipodia in Trio-deficient HeLa cells was measured. Trio-deficient cells were rescued with Trio-D1 expression and subsequently transfected with siCTRL (open bars), siRac1 (hatched bars) or siRhoG (closed bars). siRac1-treated cells showed significant decrease in the velocity (left graph), distance (middle graph) and frequency (right graph) of lamellipodia dynamics. At least nine different lamellipodia are quantified in nine different cells over three independent experiments. Data are mean ± SEM. *p<0.01.

Detailed study of the dynamics of lamellipodia by means of kymograph analyses showed that control cells formed broad and stable lamellipodia, whereas Trio-deficient cells generated thin and unstable membrane protrusions (Figure 8A). However, in Trio-silenced cells that expressed Trio-D1, the formation of lamellipodia was restored and comparable to control cells (Figure 8A). Quantification of the kymographs indicated that Trio-D1 significantly promoted the velocity, distance and frequency of the lamellipodia (Figure 8B). When compared the lamellipodial dynamics between the shCTRL and shTrio-treated cells, we found a significant decrease in lamellipodia velocity when Trio was silenced (Figure S2B). However, distance and frequency were unaltered. Expressing TrioD1 on the Trio-deficient cells promoted lamellipodial dynamics more than in shCTRL-cells (Figure S2B). We have also tested whether the SH3 domain of Trio-D1 affected the dynamics of Trio-D1-induced lamellipodia. However, using Trio-D1 mutant lacking the SH3 domain, we did not observe any significant difference in the velocity, distance or frequency of the induced lamellipodia, indicating that the SH3 domain is not required for lamellipodia dynamics (Figure 8C). To determine whether Trio-D1-induced lamellipodia dynamics were dependent on Rac1 or RhoG, we silenced these GTPases with siRNA in Trio-deficient HeLa cells (Figure 9A), which were rescued by expressing Trio-D1. Our data showed that the dynamics of lamellipodia formation in Trio-D1-rescued HeLa cells were unaltered in siCTRL or siRhoG –treated cells (Figure 9B). However, in cells that were silenced for Rac1, lamellipodia dynamics were severely impaired (Figure 9B). Kymograph analysis underscored these observations and showed a significant reduction in lamellipodia velocity, distance and frequency when Rac1 was depleted (Figure 9C). Thus, these data show that the Trio-D1 GEF domain induces the dynamics of lamellipodia in a Rac1-dependent manner and is required for fibronectin-mediated cell spreading and migration.

## Discussion

The Rac1/RhoG/RhoA GEF Trio is ubiquitously expressed and plays an important role in neurite outgrowth and secretion of mediators from granules in neuronal cells [13], [21], [22]. However, not much is known about the cellular functions of Trio and the role of its different GEF domains. We show here that the N-terminal DH-PH domain of Trio (Trio-D1) activates Rac1 independently from RhoG in cell cultures. In fact, we observed increased Rac1 activation by Trio-D1 when RhoG expression was silenced. This suggests that Rac1 competes with RhoG for binding to Trio-D1. Although it is well recognized that the N-terminal DH-PH unit of Trio is required for the activation of GTPases, less is known about the flanking SH3 domain. We show here that the SH3 domain is required for Trio-D1 binding to the proline-rich C-terminus of Rac1, but not of RhoG. It has been shown by Yohe and co-workers that the SH3 domain of NGEF and WGEF acts as an auto-inhibitory signal by sterically blocking the DH-domain [23]. However, deleting the SH3 domain of Trio-D1 did not affect its exchange activity for Rac1 or RhoG, indicating that the SH3 domain is not auto-inhibitory. Interestingly, we noticed that the nucleotide-free RhoG but not Rac1 mutants bound less efficiently to Trio-D1 when the SH3 domain was deleted. These nucleotide-free mutants have a high affinity for GEFs in their activated state, indicating that the SH3 domain influences the activity state of Trio. Also, these data show that the SH3 domain is more involved in the binding of Trio to the GTPase and not so much in the activation, since expression of Trio-D1 lacking the SH3 domain still induces Rac1 and RhoG activation. Thus, the SH3 domain that flanks the N-terminal DH-PH domain of Trio promotes the exchange between GDP and GTP on Rac1 and RhoG, but is not essential. Based on this conclusion, one may predict that Trio-D1 lacking the SH3 domain is also less efficient in activating Rac1 or RhoG, as measured with classical pull-down assays. However, using these assays, we did not find significant differences in Rac1 or RhoG activation upon expression of Trio-D1, in the absence or presence of the SH3 domain. The expected effect of the SH3 domain on Trio-induced Rac1 and RhoG activity may be moderate and therefore easily overlooked, because of the ectopic expression system that we use. Seipel and colleagues showed that the Trio-N-terminal SH3 domain is involved in colony formation in soft agar [14]. However, these assays take 18 days of culture, which may indicate that the SH3 domain- mediated effects are subtle and require time to become apparent. Others reported that the SH3 domain is involved in neurite outgrowth [21]. Also these assays take more than 2 days in total. Therefore, the effects of the SH3 domain of Trio-D1 on Rac1 and RhoG activation in our expression system with cultured HeLa cells may be not sensitive enough to pick up the differences that we observed with the nucleotide-free mutants.

Rac1 and RhoG signaling has been implicated in cell spreading by several groups [5], [7]. However, a role for RhoG in cell spreading is controversial, because others have shown that depletion of RhoG did not affect cell spreading or Rac1 activation [8]. Our personal observations show that cell spreading was not affected upon silencing of RhoG expression (MH, JDvB). The involvement of RhoGEFs in cell spreading is less well understood. Some RhoGEFs, such as p115RhoGEF and LARG, have been implicated in spreading, but others, such as Ect2 or Dbl have been excluded [24]. Dubash and colleagues showed that these GEFs are responsible for RhoA activation [24]. Arthur and co-workers found that the RacGEFs Tiam1 and Vav2, but not β-Pix or SWAP-70 are involved in cell spreading [25]. These GEFs are recruited to the plasma membrane, where they are activated and locally exchange GDP for GTP on Rac1. In addition, DOCK180 has also been implicated in cell spreading, since silencing this a-typical GEF results in impaired Rac1 activation upon epithelial cell spreading onto fibronectin [8]. For Trio, the group of Streuli showed that overexpression of Trio-D1 promoted the formation of lamellipodia [14]. Using kymograph analysis on Trio-silenced cells that were rescued with Trio-D1 expression, we showed that Trio-D1 promotes the induction of lamellipodia, required for proper cell migration [26]. Interestingly, our data indicated that Trio-D1 acts through Rac1 when mediating lamellipodia formation. These data were obtained in HeLa as well as Cos7 cells (data not shown). This is in line with the data we obtained when measuring the influence of Trio on GTPase activity during cell spreading on fibronectin. Only Rac1, and not RhoA or RhoG activity was impaired in Trio-deficient cells upon spreading. Others have shown that RhoG is a major downstream effector GTPase of Trio-D1 unit [12]. However, these data were obtained with dominant-negative mutants. Our approach was based on protein silencing. Trio silencing results in instable lamellipodia, comparable as is observed in Trio-deficient neurite growth cones [27]. Therefore, based on the above described data, we conclude that Rac1, and not RhoG, acts as the major GTPase downstream of Trio-D1. Altogether, these data indicate that Trio is a regulatory GEF that promotes integrin-mediated cell spreading through its N-terminal DH-PH unit by regulating GTP exchange on the small GTPase Rac1.

## Materials and Methods

### Materials

Monoclonal antibodies (mAb) to Rac1, beta-catenin and paxillin were purchased from Transduction Laboratories (Becton Dickinson, Amsterdam, The Netherlands). RhoA mAb, p-JNK Ab and Trio polyclonal Ab (clone D20) were purchased from Santa Cruz Biotechnology. RhoG mAb was a kind gift from Dr. J. Meller and Dr. M.A. Schwartz, University of Virginia, Charlottesville, VA, USA). Anti-HA mAb, Texas-Red- or Alexa-633- or Alexa-405-Phalloidin, Alexa-488-labeled GαM-Ig, Alexa-568-labeled GαM-Ig and Alexa-568-labeled GαR-Ig secondary Abs were purchased from Invitrogen (Leiden, The Netherlands). The GFP and Myc (clone 9E10) mAbs were purchased from Invitrogen (Carlsbad, CA, USA). Actin mAb was purchased form Sigma-Aldrich (Zwijndrecht, the Netherlands).

### Cell Culture

HeLa cells were cultured in IMDM (Invitrogen) supplemented with 10% (v/v) heat-inactivated fetal calf serum, 1% glutamine and antibiotics and kept at 37°C at 5% CO_2_, as previously described by Nethe et al. [10]. Cells were transfected by means of *Trans*IT®-LT1 Transfection Reagent (Mirus Bio, Madison, WI, USA).

### RhoG and Rac1 knockdown

siRNA duplexes against human RhoG (sense, 5′-P-GCAACAGGAUGGUGUCAAGUU- 3′; antisense, 5′-P-UCGUCCAAGAUCGACAUCCUU- 3′), siRac1, described by De Kreuk et al [28] and siControl non-targeting siRNA were obtained from the Dharmacon siRNA collection. HeLa cells were transfected with 50 nmol/l siRNA by means of INTERFERin transfection reagent (Polyplus, Illkirch, France). After 48 h, cells were processed for experiments.

### Stable Trio knockdown cells

HeLa cells with stable Trio knockdown through the SilencX technology were purchased from Tebu-Bio (Heerhugowaard, the Netherlands). To test stable Trio silencing, Trio protein expression was routinely tested using pre-cast 3–8% gels (Invitrogen). As a control, scrambled shRNA sequence was used and stably expressed in HeLa cells.

### cDNA plasmids

GFP-Trio-D2 was generated by cloning Trio-D2 from HA-Trio-D2 with primers JR2F 5′-GAGATCCTCGAGCTGAGCTCGTCAGTGCAATTG-3′ and JR2R 5′-GAGATCGAATTCCTAGGCTGCAGAGGAGACCAG-3′ into the pEGFP C1 vector using XhoI and EcoRI restriction sites. GFP-Trio-D1 and GFP-Trio-D1ΔSH3 were generated by cloning TrioD1 or Trio-D1ΔSH3 from Myc-Trio FL with primers JR1F 5′-GAGATCCTCGAGCTAGGAAAGAGTTCATAATGGCT-3′ and JR4R 5′-GAGATCGAATTCCTAGGCGTCATTGCTGGAGAC-3′ or JR3R 5′- GAGATCGAATTCCTACTTAGGGATGTGAATGGGC-3′, respectively, into the peGFP C1 vector with XhoI and EcoRI restriction sites.

### Cell spreading

Cells were harvested and washed 3 times in serum-free IMDM medium. Cells were kept in suspension for 30 minutes at 37°C, 5% CO_2_. Next, cells were plated on FN-coated surfaces in serum-free IMDM medium as indicated and subsequently fixed for immuno-fluorescence imaging analysis or biochemical analysis.

### Cell migration

Cells were kept in suspension for 30 minutes at 37°C at 5% CO_2_ in serum-free conditions. Next, cells were added to the upper compartment of a Transwell filter and allowed to migrate through the filter pores (8 µm) to the other side for 5 h. The filter was coated with 10 µg/mL FN. Cells were removed from the upper compartment with a cotton swap and cells on the bottom side were stained with DAPI to mark the nuclei. By confocal imaging and Image J (NIH), the number of nuclei per field of view was quantified.

### Scanning electron microscopy

Transfected cells were grown on glass coverslips, fixed in McDowell's fixative for 30 minutes at room temperature, and processed for scanning electron microscopy as described previously [29]. Cells were examined on a scanning electron microscope (model 820; JEOL Corp., Peabody, MA, USA) at 15 kV.

### Immuno-fluorescence imaging

HeLa cells were plated on FN-coated glass cover slips for the indicated times and subsequently fixed and immuno-stained as described [30]. Staining was followed by secondary staining with fluorescently labeled Abs. F-actin was visualized by Phalloidin staining. Images were recorded with a ZEISS LSM510 confocal microscope with appropriate filter settings. Cross-talk between different channels was avoided by use of sequential scanning. The dynamics of membrane ruffles were analyzed on kymographs generated from the time-lapse movies with ImageJ (NIH).

### RhoA, RhoG and Rac1 pull down assay

After lysis of the cells (25 mM Tris, 150 mM, NaCl, 10 mM MgCl_2_, 2 mM EDTA, 0.02% SDS, 0.2% Deoxycholate, 1% Triton X-100, pH 7.4) and centrifugation (10 minutes at 14,000 g) of the cytoskeletal fraction, C21-Rhotekin-GST fusion protein (for active RhoA), according to Ren et al. [31], or biotin-tagged CRIB peptide (for active Rac1), as described previously [10] or GST-ELMO (GST fusion protein containing the full-length RhoG effector ELMO) conjugated to glutathione–Sepharose beads (GE Healthcare, Zeist, The Netherlands), as described previously [6], was added to the supernatant. After incubation for 30 minutes at 4°C, GST-beads were centrifuged, washed and analyzed by SDS-PAGE.

### Fusion proteins

GST-ELMO, RhoG-G15A and GST-Rac1-G15A fusion proteins were purified from BL21 *Escherichia coli* cells (Agilent technologies, Amstelveen, The Netherlands) with glutathione–Sepharose 4B as previously described [6]. GST fusion proteins were stored in 30% (v/v) glycerol at −80°C. The pull down experiment for the Rac1/RhoG-G15A mutants was performed using the same method as for the GTPase pull down assays, as described above.

### Peptide pull-down assays

Peptide pull-down assays were performed as described previously [9]. In short, each assay was performed with 5 µg of indicated biotin-labeled peptide and 25 µl Streptavidin-coated beads (Sigma-Aldrich) in lysis buffer (25 mM Tris, 150 mM, NaCl, 10 mM MgCl_2_, 2 mM EDTA, 0.02% SDS, 0.2% Deoxycholate, 1% Triton ×100, pH 7.4).

### Peptide synthesis

Peptides were synthesized on a peptide synthesizer (Syro II) by means of Fmoc solid-phase chemistry. Peptides encoded a biotinylated protein transduction domain (biotin-YARAAARQARAG) [32] followed by the 10 amino acids preceding the CAAX domain for all Rho GTPase peptides used. The sequences of Rac1 (P-A) and the Rac1 (RKR-AAA) mutants are CAAAVKKRKRK and CPPPVKKAAAK, respectively.

### Western blot analysis

Samples were analyzed by SDS-PAGE. Proteins were transferred to 0.45-µm nitro-cellulose sheets (Schleicher and Schnell Inc., USA) and subsequently incubated with the appropriate Abs overnight and finally developed with an enhanced chemiluminescence (ECL) detection system (Amersham).

### Electric Cell-substrate Impedance Sensing (ECIS)

HeLa cells were added at 100,000 cells per well (0.8 cm^2^) to a FN-coated electrode-array and allowed to spread in serum-free conditions. The resistance was measured on line at 37°C at 5% CO_2_ with the ECIS-Model-Z Theta Controller from BioPhysics, Inc. (Troy, NY, USA). After 6–8 h, data were collected and changes in resistance of the monolayer were analyzed.

### Kymograph analysis

Lamellipodia dynamics with statistical significance by means of kymograph analysis were described by Hinz et al [33]. Briefly, protruding and retracting membrane ruffles were manually tracked with ImageJ. Specific parameters of a lamellipodium were the velocity, in um per minute, persistence in minutes and the frequency, in ruffle per minute. At least 9 different cells were analyzed from three independent experiments. Of each cell, at least two different lamellipodia were analyzed.

### Statistics

Student's t-test for paired samples (two-tailed) was used where indicated.

## Supporting Information

Figure S1 **Trio does not affect RhoA inactivation upon spreading on fibronectin.** Cells were allowed to spread on FN for indicated times in minutes. RhoA activity was measured with G-LISA according to manufacturer's protocol (Cytoskeleton Inc, Denver, CO). Data show that RhoA activity was high in suspended cells and decreased in time upon spreading. No difference between shCTRL and shTrio cells was measured.
(TIF)

Figure S2 **(A) Expression of Trio-D2 does not rescue defect in cell spreading.** HeLa cells with a stable knock down for Trio (shTrio) were allowed to spread on fibronectin-coated surfaces under serum-free conditions. Cells were transfected with either GFP-Trio-D1 or GFP-Trio-D2 (green), as indicated. Image analysis by confocal microscopy showed that Trio-D1, but not Trio-D2 rescued the spreading defect induced by Trio silencing. Bar, 20 µm. (B) Kymograph analysis as described in Materials and Methods section of the dynamics of lamellipodia in control HeLa cells, Trio-deficient HeLa cells and Trio-deficient cells that were rescued with Trio-D1 expression. Trio-deficient cells showed significant decrease in the velocity (left graph), but not in the distance (middle graph) and frequency (right graph) of lamellipodia dynamics compared to control cells. Rescuing TrioD1 activity in Trio-deficient cells did promote lamella velocity, distance and frequency significantly compared to Trio-deficient cells. At least nine different lamellipodia are quantified in nine different cells over three independent experiments. Data are mean ± SEM. **p<0.01, *p<0.05.
(TIF)
