# Supplementary figures and images for: The N-Terminal DH-PH Domain of Trio Induces Cell Spreading and Migration by Regulating Lamellipodia Dynamics in a Rac1-Dependent Fashion
Source: PLoS One. 2012 Jan 6;7(1):e29912. doi: 10.1371/journal.pone.0029912 (PMC3253119; doi:10.1371/journal.pone.0029912)

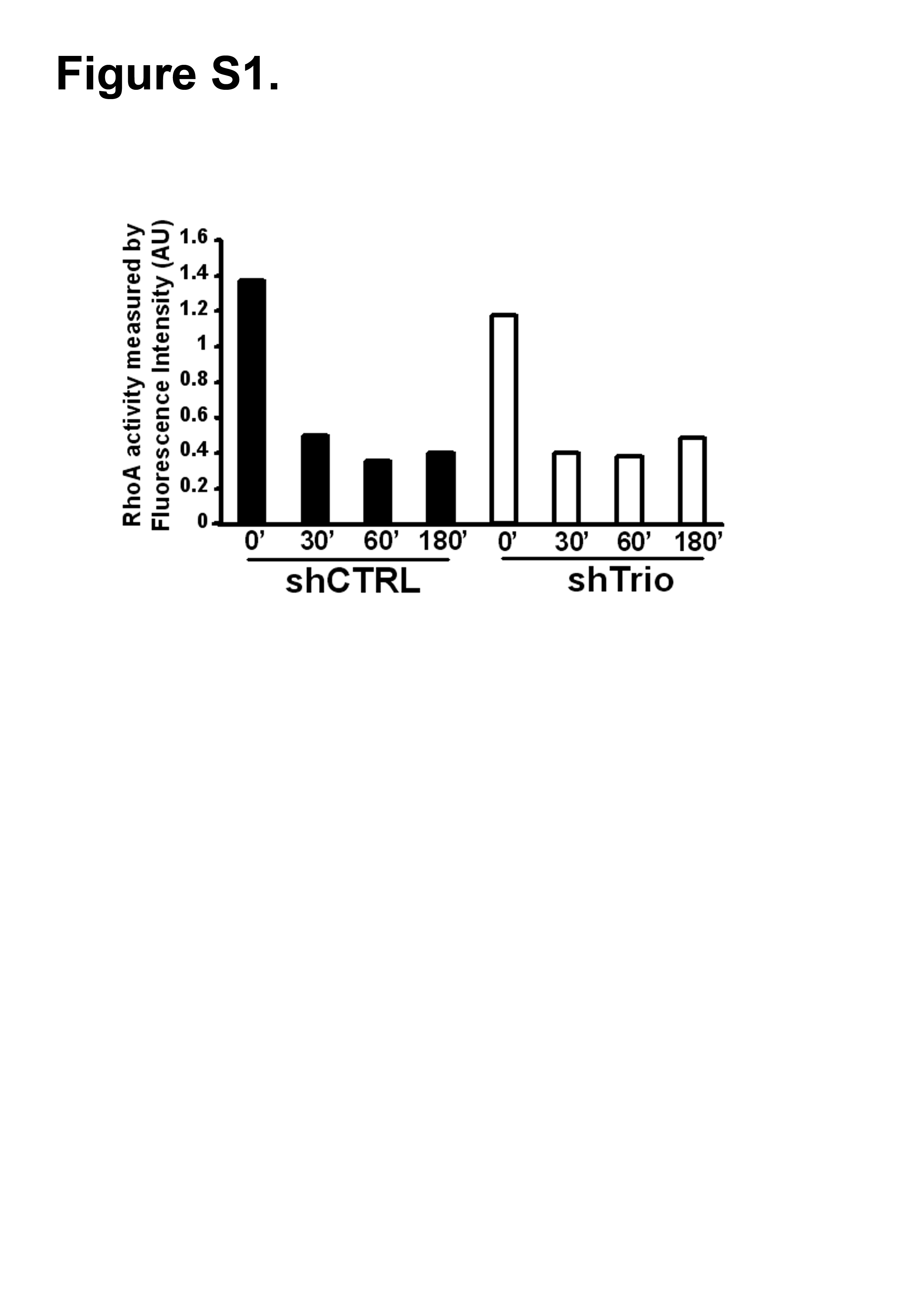

Supplement: Figure S1 — Trio does not affect RhoA inactivation upon spreading on fibronectin. Cells were allowed to spread on FN for indicated times in minutes. RhoA activity was measured with G-LISA according to manufacturer's protocol (Cytoskeleton Inc, Denver, CO). Data show that RhoA activity was high in suspended cells and decreased in time upon spreading. No difference between shCTRL and shTrio cells was measured. (TIF) [file pone.0029912.s001.tif]

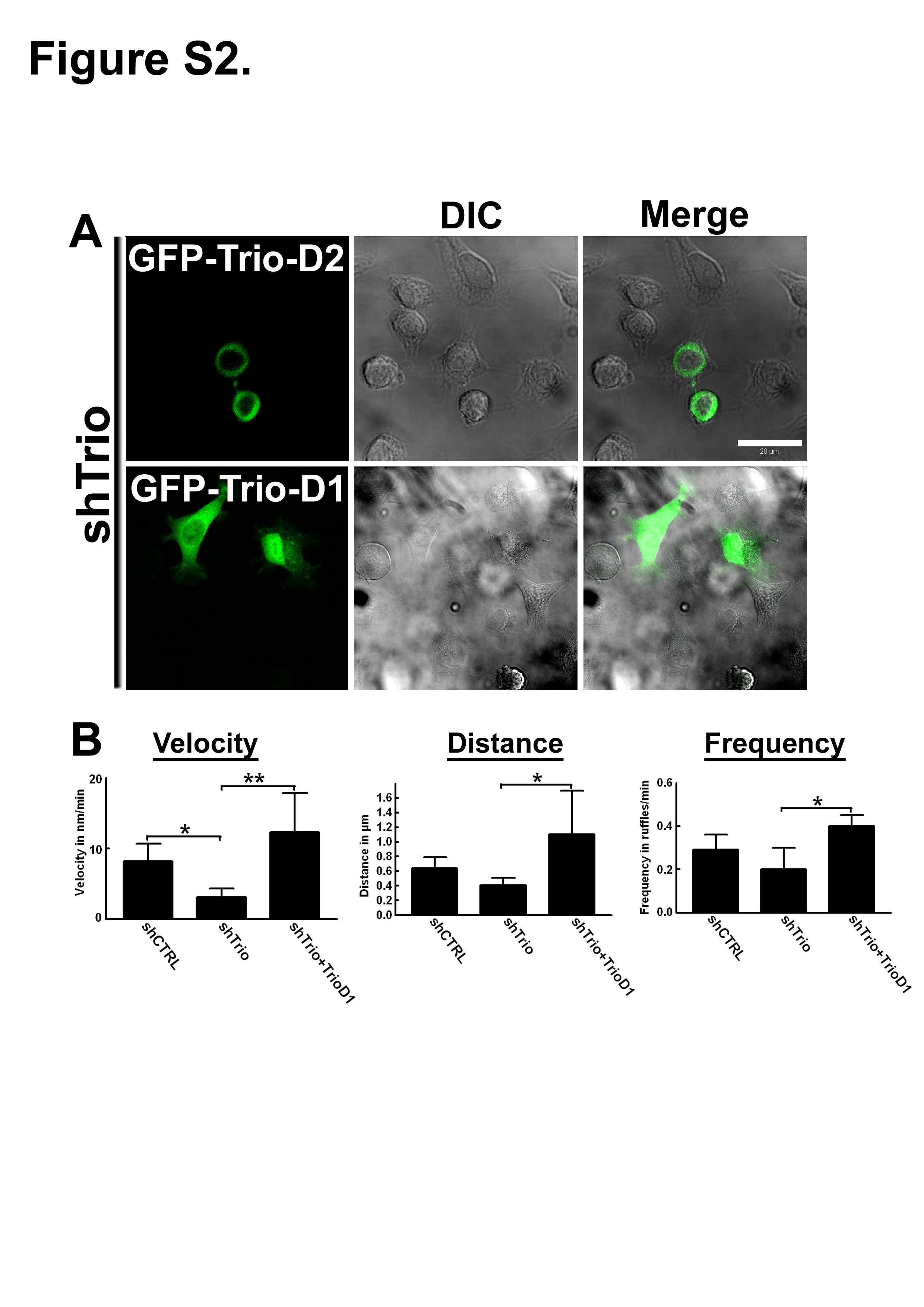

Supplement: Figure S2 — (A) Expression of Trio-D2 does not rescue defect in cell spreading. HeLa cells with a stable knock down for Trio (shTrio) were allowed to spread on fibronectin-coated surfaces under serum-free conditions. Cells were transfected with either GFP-Trio-D1 or GFP-Trio-D2 (green), as indicated. Image analysis by confocal microscopy showed that Trio-D1, but not Trio-D2 rescued the spreading defect induced by Trio silencing. Bar, 20 µm. (B) Kymograph analysis as described in Materials and Methods section of the dynamics of lamellipodia in control HeLa cells, Trio-deficient HeLa cells and Trio-deficient cells that were rescued with Trio-D1 expression. Trio-deficient cells showed significant decrease in the velocity (left graph), but not in the distance (middle graph) and frequency (right graph) of lamellipodia dynamics compared to control cells. Rescuing TrioD1 activity in Trio-deficient cells did promote lamella velocity, distance and frequency significantly compared to Trio-deficient cells. At least nine different lamellipodia are quantified in nine different cells over three independent experiments. Data are mean ± SEM. **p<0.01, *p<0.05. (TIF) [file pone.0029912.s002.tif]
